# Gene discovery for the carcinogenic human liver fluke, *Opisthorchis viverrini*

**DOI:** 10.1186/1471-2164-8-189

**Published:** 2007-06-22

**Authors:** Thewarach Laha, Porntip Pinlaor, Jason Mulvenna, Banchob Sripa, Manop Sripa, Michael J Smout, Robin B Gasser, Paul J Brindley, Alex Loukas

**Affiliations:** 1 Department of Parasitology, Khon Kaen University, Khon Kaen, Thailand; 2 Department of Pathology, Khon Kaen University, Khon Kaen, Thailand; 3 Division of Infectious Diseases and Immunology, Queensland Institute of Medical Research, Brisbane, Australia; 4 Department of Veterinary Science, University of Melbourne, Melbourne, Australia; 5 Department of Tropical Medicine, Tulane University Health Sciences Center, New Orleans, USA

## Abstract

**Background:**

Cholangiocarcinoma (CCA) – cancer of the bile ducts – is associated with chronic infection with the liver fluke, *Opisthorchis viverrini*. Despite being the only eukaryote that is designated as a 'class I carcinogen' by the International Agency for Research on Cancer, little is known about its genome.

**Results:**

Approximately 5,000 randomly selected cDNAs from the adult stage of *O. viverrini *were characterized and accounted for 1,932 contigs, representing ~14% of the entire transcriptome, and, presently, the largest sequence dataset for any species of liver fluke. Twenty percent of contigs were assigned GO classifications. Abundantly represented protein families included those involved in physiological functions that are essential to parasitism, such as anaerobic respiration, reproduction, detoxification, surface maintenance and feeding. GO assignments were well conserved in relation to other parasitic flukes, however, some categories were over-represented in *O. viverrini*, such as structural and motor proteins. An assessment of evolutionary relationships showed that *O. viverrini *was more similar to other parasitic (*Clonorchis sinensis *and *Schistosoma japonicum*) than to free-living (*Schmidtea mediterranea*) flatworms, and 105 sequences had close homologues in both parasitic species but not in *S. mediterranea*. A total of 164 *O. viverrini *contigs contained ORFs with signal sequences, many of which were platyhelminth-specific. Examples of convergent evolution between host and parasite secreted/membrane proteins were identified as were homologues of vaccine antigens from other helminths. Finally, ORFs representing secreted proteins with known roles in tumorigenesis were identified, and these might play roles in the pathogenesis of *O. viverrini*-induced CCA.

**Conclusion:**

This gene discovery effort for *O. viverrini *should expedite molecular studies of cholangiocarcinogenesis and accelerate research focused on developing new interventions, drugs and vaccines, to control *O. viverrini *and related flukes.

## Background

Throughout East Asia, there is a strikingly high prevalence of cholangiocarcinoma (CCA – cancer of the bile ducts) in regions where the human liver fluke is endemic. No stronger link occurs between a human malignancy and infection with a eukaryotic parasite than that between CCA and infection with the liver fluke, *Opisthorchis viverrini *(Digenea) [1]. Indeed, the International Agency for Research on Cancer (IARC) recognizes *O. viverrini *as a 'category I carcinogen' [2,3]. CCA is highly prevalent in Northeast Thailand, areas where uncooked cyprinoid fish are a dietary staple. Due to poor sanitation practices and inadequate sewerage infrastructure, *O. viverrini*-infected people pass the trematode's eggs in their feces into natural bodies of fresh water. Aquatic snails, which represent the first intermediate hosts of *O. viverrini*, ingest the eggs from which the miracidia undergo asexual reproduction before a population of the free swimming larval stage, called a cercaria, is shed from the infected snails. The cercaria then locates a cyprinoid fish, encysts in the fins, skin and musculature of the fish, and becomes a metacercaria. The metacercarial stage is infective to humans and other fish-eating mammals. Infection is acquired when people ingest raw or undercooked fish. The young adult worm escapes from the metacercarial cyst in the upper small intestine and then migrates through the ampulla of Vater into the biliary tree, where it develops to sexual maturity over four to six weeks, thus completing the life cycle. The adult worms, which are hermaphrodites, can live for many years in the liver, even decades, shedding as many as 200 eggs per day which pass out via bile into the chyme and feces [4].

In Thailand, ~6 million people are infected with *O. viverrini*. Despite widespread chemotherapy with the compound, praziquantel, the prevalence of *O. viverrini *in some endemic areasapproaches 70% (reviewed in [1]). Moreover, in Thailand, liver cancer is the most prevalent of the malignant/fatal neoplasias, and the prevalence of CCA in regions in which the parasite is endemic is unprecedented [5].

While sexual reproduction takes place in the mature adults of *O. viverrini *within the bile ducts, asexual reproduction in the snail leads to a massive increase in the number of infectious cercarial stages exiting and swimming off to locate then infect the fish host. The adult fluke is a diploid organism which reproduces by meiosis; self fertilization of the male and female organs occurs, but it is believed that cross-fertilization between adjacent adult worms is the normal pattern. Although the genome size of *O. viverrini *has not yet been reported, it is known to have six pairs of chromosomes, i.e. 2n = 12 [6], distinct from the closely related liver fluke, *Clonorchis sinensis*, which possesses 2n = 56 chromosomes [7].

Despite its public health importance, only a small number of *O. viverrini *sequences (mostly ribosomal genes) have been available in public databases prior to the present study. Characterization of the genes expressed in this organism should provide a foundation for elucidating the immunopathogenesis of CCA, particularly the molecular mechanisms by which infection with this parasite induces cancer. Indeed, secreted proteins of *O. viverrini *induce hyper-proliferation of cells (or hyperplasia) *in vitro *[8], implying that carcinogenesis may not be just a consequence of chronic inflammation, but that the parasite actively secretes gene products which initiate neoplasia.

Here, we undertake gene discovery for *O. viverrini *after the construction of a cDNA library and characterization of ~5,000 expressed sequence tags (ESTs) from this carcinogenic parasite. A similar dataset exists for *C. sinensis *[9], which, despite its widespread prevalence [10], is not recognized as a 'class I carcinogen' [3]. Therefore, we compared the available transcriptomic dataset from *O. viverrini *with those from *C. sinensis*, and from several other flatworms, both free-living and parasitic in humans.

## Results and Discussion

### Features of the dataset

Of 5,159 randomly selected ESTs, a total of 4,241 yielded acceptably high quality sequences. These in turn were clustered into contigs, establishing a catalogue of 1,932 non-redundant OvAEs. This apparently represents the largest dataset thus far for any of the liver flukes. Table 1 summarizes the key features of the dataset. Of note is that the identities for 1,070 (55%) of these OvAEs could not yet be established, as they did not share sequence homology (BLASTx/tBLASTx) with any other predicted or known molecules in public databases, including dbEST which contains 2,678 ESTs from the related liver fluke, *C. sinensis *[9]. The average insert size of these novel OvAEs was 550 nt; 47 of these 1,070 OvAEs had insert sizes of less than 150 nt. These ESTs may in fact be *O. viverrini*-specific or even digenetic fluke-specific genes. A similar situation currently pertains to the human blood fluke where a large percentage of known transcripts, and indeed proteins, are assumed to be *Schistosoma*- or indeed phylum Platyhelminthes-specific [11,12]. If the *O. viverrini *genome has 14,000 protein-coding genes (like the blood fluke *S. mansoni*) [13], and if each of the 1,932 *O. viverrini *contigs represented a protein coding gene, these newly discovered genes from the adult stage of *O. viverrini *are predicted to represent ~14% of the entire transcriptome of this liver fluke. EST sequences described herein have been deposited in dbEST under accession numbers EL618683–EL620614.

**Table 1:** Features of the *Opisthorchis viverrini *EST catalogue.

**Feature**	**Number**
Initial Sequences	5159
Usable sequences^a^	4241
Contigs	1995 (1632 singletons; 363 clusters)
Contigs after clean-up^b^	1932
Contigs identical to known proteins^c^	68
Contigs similar to other proteins^d^	794
Contigs with gene ontology assignments	383
Novel contigs	1070
Novel contigs with signal sequences	75 (29 signal peptides; 46 signal anchors)
Average insert size	548 bp (ESTs); 660 bp (contigs)
Percentage of recombinant clones	95%
Number of ribosomal seqs	1184 ESTs; 136 clusters

^a^Usable sequences were determined using seqclean – sequences that were removed were either non-recombinant, of low complexity and/or quality and those of < 100 nt in length.

^b^clean-up refers to removal of sequences from contaminating sources; eg. *Mycoplasma*

^c^identity determined by ≥ 95% identity over ≥ 50 amino acids.

^d^based on BLASTx and tBLASTx searchers of GenBank nr and dbEST respectively.

### Abundantly expressed transcripts

After manual filtering of 136 ribosomal sequences, the 10 most abundantly represented mRNAs encoded proteins with known or unknown functions, including one contig that did not have homologues in any public databases (Table 2). Abundant contigs encoded proteins involved in a range of physiological functions which are considered essential to parasitism, such as anaerobic respiration (myoglobin) [14], reproduction (vitelline precursors and egg shell proteins) and detoxification of xenobiotic compounds (glutathione-*S*-transferase). Other abundantly expressed OvAEs encoded proteins of likely key relevance to the host-parasite relationship, and included proteases (papain-like and legumain-like enzymes), saposin-like proteins and dynein light chains. Homologues of some of the most abundantly represented OvAEs were also highly represented in *C. sinensis *ESTs (cysteine proteases, myoglobin, vitelline B precursor), whereas others were uniquely over-expressed in each species. In particular, structural molecules, including tubulin and actin-binding proteins, were among the 10 most abundant clones from *C. sinensis *[9], but were not highly represented in the dataset from *O. viverrini*. An in-depth comparison of the *O. viverrini *and *C. sinensis *datasets is presented below.

**Table 2:** The 10 most abundant contigs^a ^from the *Opisthorchis viverrini *EST dataset.

**Contig**	**ESTs/contig**	**Closest homologue in GenBank nr (accession no.)**	**%identities (no. of aa)**	**Score (Bits)**	**Closest homologue in dbEST (accession no.)**	**%identities (no. of aa)**	**Score (Bits)**
OvAE1587	100	vitelline B precursor, *O. viverrini *(AAL23712)	99% (230)	493	*C. sinensis *cDNA clone CSAD-01-D02 (AT007557)	92% (225)	524
OvAE1588	77	17 kDa myoglobin, *Clonorchis sinensis *(AAM18464)	81% (149)	244	*C. sinensis *cDNA clone CSAD-29-A12 (AT009373)	77% (188)	344
OvAE1585	77	hypothetical protein, *C. sinensis *(AAM55183)	84% (90)	156	*C. sinensis *cDNA clone CS30 (AT006763)	84% (100)	199
OvAE1593	41	egg protein, *C. sinensis *(AAN64160)	89% (253)	389	*C. sinensis *cDNA clone CSAD-20-B05 (AT008604)	82% (237)	477
OvAE1584	37	hypothetical protein, *Macaca fascicularis *(BAE73006)	67% (59)	82	SJA_AAF_D11.T3 SJA *S. japonicum *(CX857852)	85% (94)	183
OvAE1602	21	histone H1, *Schistosoma japonicum *(AAP06509)	74% (70)	112	*C.sinensis *cDNA clone CSAD-25-H03 (AT009091)	85% (177)	302
OvAE1607	17	egg protein, *C. sinensis *(AAN64160)	59% (252)	288	*C. sinensis *cDNA clone CSAD-01-B01 (AT007532)	82% (252)	493
OvAE1595	16	retrotransposon gag region, *Monascus pilosus *(ABC24965)	31% (57)^b^	33	NA	NA	NA
OvAE1608	15	translationally controlled tumor protein, *C. sinensis *(AAX84199)	98% (169)	306	*C. sinensis *cDNA clone CSAD-24-E07(AT008979)	92% (122)	228
OvAE1601	11	glutathione-*S*-transferase, *O. viverrini *(AAL23713)	98%(213)	429	*C. sinensis *cDNA clone CSAD-32-E05 (AT009695)	86% (232)	484

^a^Non-ribosomal sequences only were used in this analysis.

^b^Sequence identity was low but diagnostic motifs of gag were detected over 57 amino acids.

### Gene ontology assignments of ESTs from *O. viverrini *and related flukes

**Figure 1:**
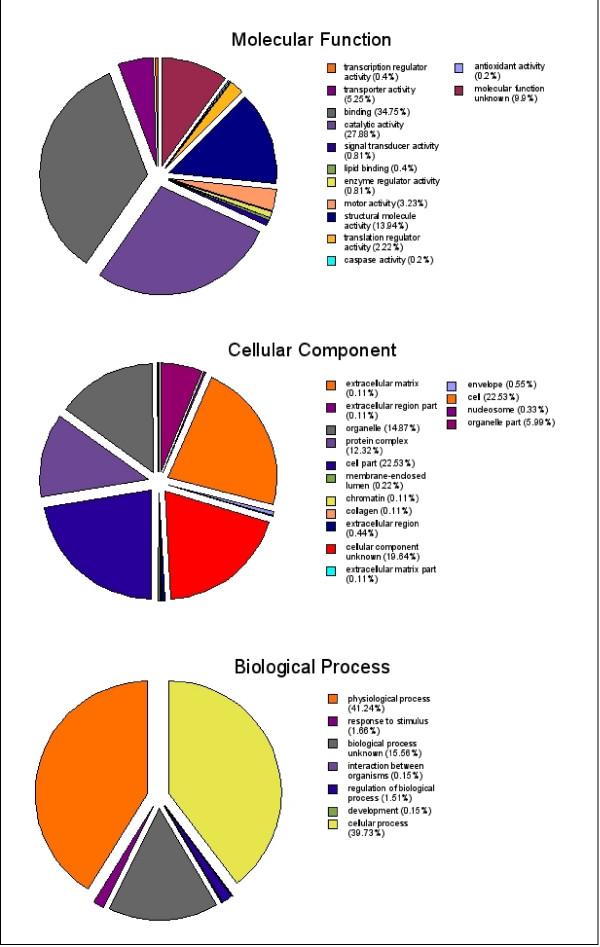
**Summary of predicted gene product function and location using gene ontology terms**. Gene ontology (GO) terms for annotated *Opisthorchis viverrini *assembled ESTs were extracted, if present, from the GO database and sorted into the immediate subcategories for molecular function, cellular component and biological process. The GO subcategory and percentage relative to the total number of extracted terms is indicated in the legend. Although cellular and physiological processes, structural proteins and catalytic activity were strongly represented other categories of interest include the caspases and transporter activity that may represent proteins important for a parasitic lifestyle. The large number of unknowns in each of the three categories highlights the lack of knowledge regarding many of the proteins found in these parasites.

Three hundred and eighty three (383) of the total 1,932 OvAEs (19.8%) could be assigned GO classifications (Figure 1). The most abundant groups represented under the molecular function category were linked to binding (34.8%), catalytic activity (27.9%) and structural molecule activity (13.9%). Other sequences of interest identified in this category were inferred to relate to caspase activity (0.2%) and transporter activity (5.3%). The most abundant groups represented under the biological process category corresponded to physiological processes (41.2%), cellular processes (39.7%) and unknown biological processes (15.6%).

**Figure 2:**
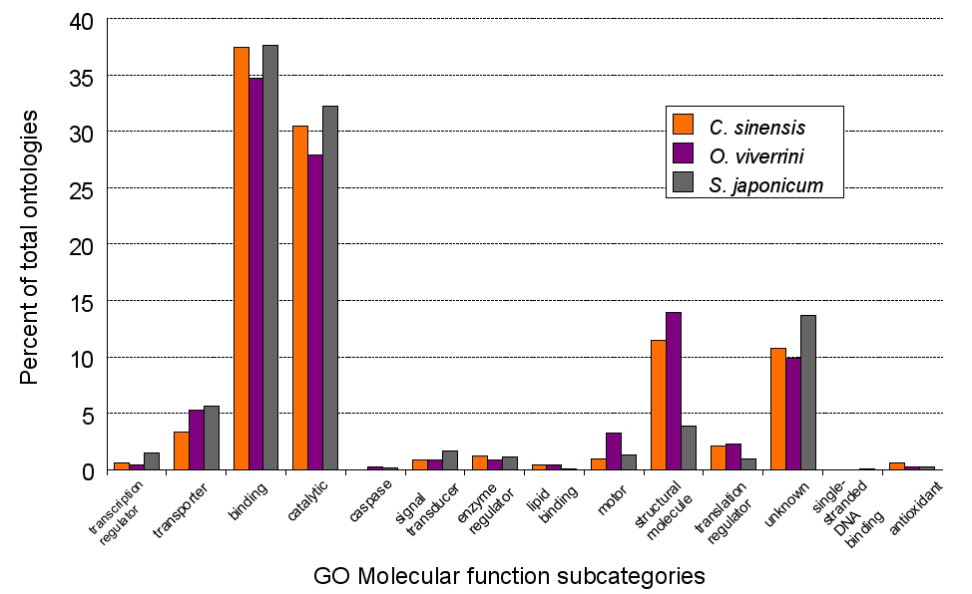
**Comparison of the gene ontology molecular function terms for expressed sequence tags from *Opisthorchis viverrini*, *Clonorchis sinensis *and *Schistosoma japonicum***. Expressed sequence tags from *C. sinensis *and *S. japonicum *were downloaded from NCBI and subjected to the same analyses used for *O. viverrini *sequences. A comparison of the percentage of terms correlating to the molecular function subcategory for each organism shows a broad similarity, although in some cases, such as categories for structural or motor proteins, categories are over- or under-represented in certain species.

We then undertook a comparative assessment of GO assignments of sequences from *O. viverrini *and two other trematode parasites of humans in Asia – the liver fluke, *C. sinensis *(2,679 contigs) and the blood fluke, *S. japonicum *(107,427 contigs). In general, the percentages of ESTs allocated to each GO category among these three flukes was similar (Figure 2). However, some categories were over- or under-represented in one species. For example, contigs encoding structural proteins were ~four times more abundantly represented in the two liver flukes than in *S. japonicum*, whereas contigs encoding motor proteins were ~three times more abundant in *O. viverrini *than they were in *C. sinensis *or *S. japonicum*. Sample sizes were too small to determine whether these differences were statistically significant. Both structural and motor proteins are important components of fluke teguments [15], playing roles in surface maintenance and turnover in schistosomes [16,17] and liver flukes [18]. Therefore, these molecular differences might reflect the specialised niches and physiological requirements of each parasite. From just 1932 OvAEs, 15 different contigs had ORFs encoding components of the dynein complex of motor proteins, a category of motion- related, and surface and gut-localized EF-hand motif- containing antigens which, at least in schistosomes, represent potent allergens and targets of protective immunological responses [17,19].

### Evolutionary relationships between *O. viverrini *and other platyhelminths

**Figure 3:**
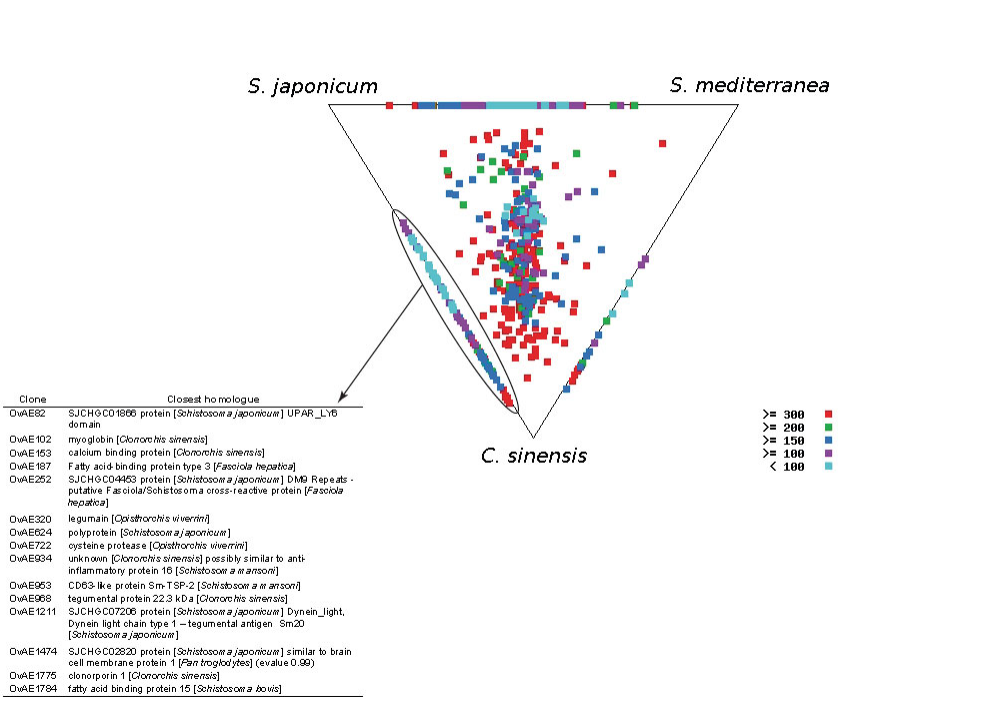
**Evolutionary relationships between *Opisthorchis viverrini *and related platyhelminths based on similarities of protein coding genes using SimiTri**. Similarity of *O. viverrini *ORFs (1,932 ESTs) to those from the liver fluke *Clonorchis sinensis *(2,679 ESTs), the blood fluke *Schistosoma japonicum *(107, 427 ESTs) and the free-living turbellarian *Schmidtea mediterranea *(171,472 ESTs). SimiTri [20] was used to plot 1,932 *O. viverrini *contigs against related species database entries (A). Each spot represents a unique contig and its sequence similarity to each of the three selected databases as determined by tBLASTx scores. Sequences showing similarity to only one database are not shown. Sequences showing sequence similarity to only two databases appear on the lines joining the two databases. Spots are coloured by their highest tBLASTx score to each of the databases. *O. viverrini *sequences with homologues in the parasitic flukes only (not in *Schmidtea*) are highlighted in the dotted region and the identities of selected examples are shown in the table (B). The entire list (105) of these putative parasite-specific proteins is shown in Table S1.

To assess the evolutionary relationships between *O. viverrini *and other platyhelminths (both parasitic and free-living), we used SimiTri [20] to plot the relative similarities of predicted polypeptide sequences (Figure 3). OvAEs shared most sequence identity with sequences from *C. sinensis *(2,679 publicly available ESTs), and *S. japonicum *(107, 427 ESTs). OvAEs were less similar to sequences from the free-living turbellarian platyhelminth, *Schmidtea mediterranea *(171,472 ESTs) [21], which was not altogether surprising given the phylogenetic distance between parasitic and free-living members of the phylum Platyhelminthes [22,23]. The bulk of this phylum (including those species analysed here) represents a monophyletic group based on 18S rDNA sequences [22] and morphological characters [24], and is often referred to as the Rhabditophora [22]. Therefore, the members of the Rhabditophora are considered to be more closely related to each other than to other turbellarian clades, such as the Polycladida [22]. A total of 105 OvAEs had homologues in the ESTs from the two parasitic flukes but not in the free-living *Schmidtea *([see additional file 1]; selected examples are shown in the table in Figure 3), suggesting that at least some of these are parasitism-specific genes. Thirty-three (33) of the conserved parasitic fluke genes were novel and did not have homologues of known function. Predicted proteins of known function included homologues of legumain, fatty acid binding proteins, myoglobin and potential anti-inflammatory proteins such as Ly6/UPAR domain-containing proteins. Of the parasitic fluke-specific genes, 38 encoded ORFs with N-terminal signal sequences; 14 of these OvAEs had homologues in just *S. japonicum *and 24 had homologues in both *S. japonicum *and *C. sinensis*.

### Secreted and membrane proteins

**Figure 4:**
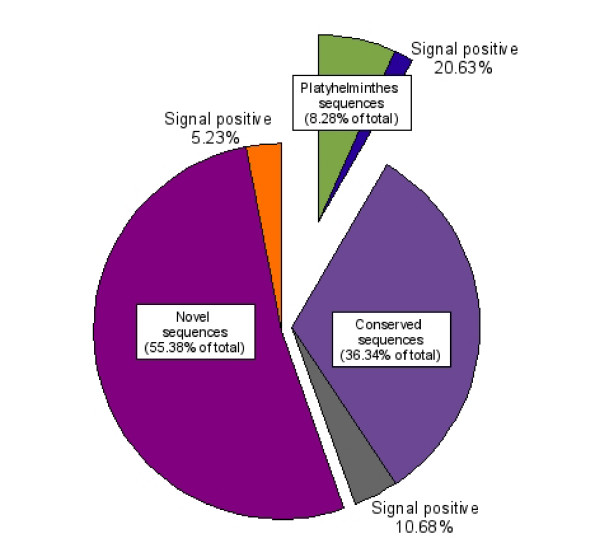
**Distribution of *Opisthorchis viverrini *assembled ESTs (OvAEs) that contain predicted signal peptides or signal anchors**. OvAEs that had BLAST hits greater than 1 × 10^-5 ^were sorted into conserved (those matching entries for species other than platyhelminths), phylum Platyhelminthes-specific (only matching platyhelminth entries) and novel (no significant homology to any database entry). The sequences in each category were then analysed for the presence of a signal sequence using SignalP. The relative percentages of each category are indicated along with the sub-category of signal sequence positive contigs.

We conducted an analysis of ORFs containing an N-terminal signal peptide or signal anchor. A total of 164 OvAEs contained ORFs with signal sequences. The dataset was divided into three categories – sequences that were (i) novel; (ii) platyhelminth-specific; (iii) conserved across multiple phyla. Novel sequences constituted 55.4% of the total, but only 5.2% of them encoded proteins with a signal sequence. Conserved sequences constituted 36.3% of the total, and 10.7% of these encoded proteins with signal sequences. Finally, the sequences inferred to be platyhelminth-specific accounted for 8.3% of the total dataset, but 20.6% of these encoded proteins with a signal sequence (Figure 4). It should be noted, however, that not all of the OvAEs contained full-length nt sequences, and therefore the true percentage of sequences with signal peptides cannot be definitively inferred in the absence of full genome coverage.

Sequences encoding novel secreted and/or membrane proteins (without orthologues/paralogues in other organisms or phyla) may be of particular interest for the development of vaccines and drugs, because the absence of host homologues enhances the prospect for therapeutic margins of safety. OvAEs encoding secreted/membrane proteins involved in many aspects of parasitism were identified (Table 3), and some of these are discussed in the following section. Two of the OvAEs presented in Table 3, which are inferred to encode transforming growth factor β receptor (see section "Host-parasite cross-talk) and calumenin, were more similar in sequence to vertebrate proteins from both the non-redundant and dbEST databases than they were to platyhelminth sequences, suggesting that they have evolved independently to bind host ligands. These results are reminiscent of reports of the schistosome transcriptome where, for example, receptors for mammalian hormones, including insulin, fibroblast growth factor and cytokines, have been hypothesized to bind host molecules (reviewed in [25]).

**Table 3:** Selected *Opsithorchis viverrini *contigs that encode families of secreted/membrane proteins that potentially interact with or are exposed to host tissues. Genera of the closest homologues from BLAST × (nr) searches are shown. Where the closest homologue was from a vertebrate (bold font), a tBLASTx search against dbEST was conducted.

**Predicted function**	**Examples/comments and genera of closest orthologues/paralogues**	**Contigs**	**%identities**
TGF-β receptor	bone morphogenic protein receptor type I (***Sus *nr/*Macaca *est**)	OvAE22	44
Seven transmembrane receptor	DC-STAMP (*Strongylocentrotus*); laminin receptor (***Bos *nr/***Clonorchis *est)	OvAE92, OvAE1722	51
Tetraspanin	stabilize cell membranes – expressed in the tegument of schistosomes (*Schistosoma*)	OvAE953	34
C1 family papain-like cysteine protease	cathepsin L (*Paragonimus*), cathepsin B (*Fasciola*; *Clonorchis*)	OvAE1795, OvAE813, OvAE1171, OvAE532, OvAE1070, OvAE1613, OvAE1711, OvAE615, OvAE398	> 80
C13 family asparaginyl endopeptidase	legumain (*O. viverrini*)	OvAE1624, OvAE1824	94
S1 family serine protease	HtrA-like (***Macaca *nr/***Schistosoma *est) and kallikrein-like (*Schistosoma*) peptidases	OvAE622, OvAE1918	47–53
A1 family aspartic protease	cathepsin D-like; digestive enzyme in helminths (*Clonorchis*)	OvAE1300	80
M41 family metalloprotease	mitochondrial membrane proteinase (*Schistosoma*)	OvAE65	91
Granulin	mitogen associated with cancer (***Bos *nr/***Clonorchis *est)	OvAE1732	45
Aquaporin	water channel protein (*Schistosoma*)	OvAE6	48
Tyrosinase	critical for *S. mansoni *egg shell production (*Schistosoma*)	OvAE1900, OvAE1854	63
Phospholipase A2	similar to vertebrate venom proteins; (***Heloderma *nr/***Clonorchis *est)	OvAE1644	55
Thioredoxin peroxidase	immunomodulatory in fasciolosis (*Schistosoma*)	OvAE54	74
EF-hand secreted Ca^2+^-binding protein	calumenin (***Rattus *nr/*Xenopus *est**)	OvAE61	47
Saposin-like protein	pore forming; similar to fluke cytolysins (*Clonorchis*)	OvAE1692	64
Pathogenesis related protein	similar to helminth venom allergen homologues (*Schistosoma*)	OvAE534, OvAE1862	38
Glutathione-S-transferase	detoxification of heme and free radicals (*Clonorchis*)	OvAE1057, OvAE1892, OvAE1601, OvAE1729	86
Synaptobrevin	neurotransmission/vesicular docking – vesicle associated (*Schistosoma*)	OvAE1001	73
Innexin	integral membrane protein forming gap junctions (*Schistosoma*)	OvAE631	78
Fibroblast growth factor (FGF) receptor substrate 2	host FGF is essential for growth of schistosomes (*Schistosoma*)	OvAE1563	32
Ly6c	Immune cell differentiation antigen	OvAE82	26

### Proteases

As with other parasitic helminth transcriptomes [11-13,25,26], proteins with catalytic activity were abundantly represented in the *O. viverrini *dataset (27.9% of contigs that were assigned GO molecular functions). Many of these enzymes encoded endo- and exo-proteases belonging to established families (MEROPS classification), but which have not yet been described from liver flukes (Table 3). Of particular interest were members of the S1A serine protease family with sequence similarity to kallikrein and chymotrypsin, and, therefore, potentially involved in feeding or tissue migration [27]. Other proteases included homologues of enzymes that digest hemoglobin in blood-feeding helminths, including cathepsin D-like aspartic and cathepsin B-like cysteine proteases [28-30] as well as an asparaginyl endopeptidase, which is known to activate the gastrodermal cathepsin B enzyme, and probably other gut proteases in *S. mansoni *[31]. We also identified *O. viverrini *homologues of the cell death enzyme, caspase-2, and the neutral cysteine protease from the tegument of schistosomes, calpain.

### Multiple membrane-spanning proteins

**Figure 5:**
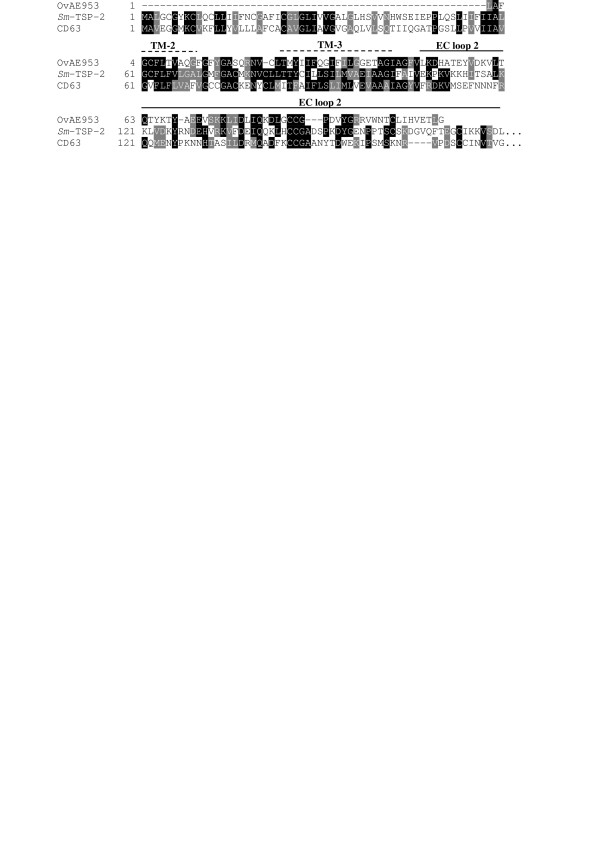
**An *Opisthorchis viverrini *homologue of *Sm*-TSP-2, a vaccine antigen expressed in the tegument of *Schistosoma mansoni***. Multiple sequence alignment comparing the ORF of OvAE953 with *Sm*-TSP-2 from *S. mansoni *(GenBank AF521091) and human CD63 (NM_001780). Both *Sm*-TSP-2 and CD63 sequences shown here are truncated at the C-terminus (fourth transmembrane domain and C-terminal tail are not shown) for comparative purposes because OvAE953 is a partial sequence. Black boxes denote identical residues shared by two or more of the sequences. Grey boxes denote conservative substitutions. Dashed lines denote the predicted transmembrane (TM) domains of *Sm*-TSP-2; the solid line represents the extracellular (EC) loop 2 region of *Sm*-TSP-2 [33].

Predicted proteins with multiple membrane spanning domains were identified. Tetraspanins, an abundantly represented family of four-transmembrane proteins in the tegument of schistosomes [32,33], were identified from *O. viverrini *(Figure 5). These proteins are thought to stabilize the cell membrane by forming a network of interactions, called the tetraspanin web, with other membrane-bound and -associated proteins, particularly on the surface of cells of the immune system [34]. A homologue of the six-transmembrane domain family of water channel proteins, aquaporin [35], was identified. Seven transmembrane proteins are common drug targets [36], and at least three distinct members of this family were identified, including receptors for dystroglycan and lamin b, and a protein with homology to a the DC-STAMP receptor from the surface of dendritic cells (Table 3).

### Host-parasite "cross talk"

**Figure 6:**
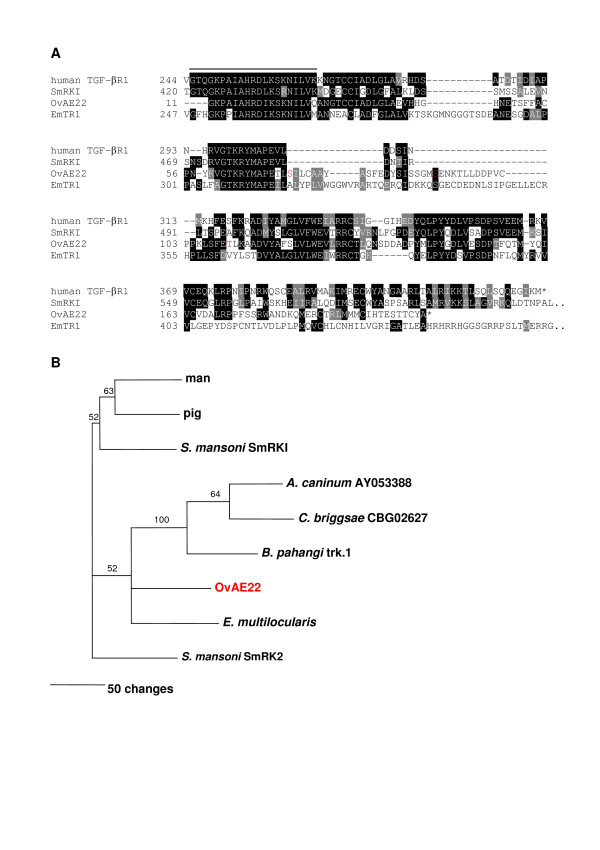
**A TGF-β receptor type I from *Opisthorchis viverrini***. Multiple sequence alignment of the ORFs of OvAE22 with homologues from *Schistosoma mansoni *(SmRK-I – GenBank AF031557), the hydatid tapeworm *Echinococcus multilocularis *(TR1 – AJ841786) and human (TGF-β receptor type I – L11695) **(A)**. The overlined region denotes the putative serine-threonine kinase active site in SmRK-I [44]. Residues highlighted in red font in OvAE22 are putative sites of serine/threonine phosphorylation. Both SmRK-I and human TGF-β receptor type I sequences shown here are truncated at the N-terminus and SmRK-I is truncated at the C-terminus for comparative purposes with the partial sequence from *O. viverrini*. Black boxes denote identical residues shared by two or more of the sequences. Grey boxes denote conservative substitutions. Neighbour joining phylogenetic tree showing the relationship between the ORF of OvAE22 and other members of the TGF-β receptor type I family **(B)**. Numbers on branches denote bootstrap values from 100 samplings. The nominated outgroup was the type 2 receptor, SmRK-2. GenBank accession numbers not already provided above are as follows: pig bone morphogenic protein (BMP) receptor type I (AY065994); dog hookworm *Ancylostoma caninum *S/T kinase (AY053388); *Caenorhabditis briggsae *CBG02627 (CAAC01000012); filarial nematode *Brugia pahangi *trk-1 (AF013991); *S. mansoni *SmRK-2 (AY550912).

Parasitic helminths receive host-derived signals for growth and reproduction *via *surface receptors for host ligands, [37-39]. Convergent evolution of extracellular parasite proteins to promote their interactions with host tissues is well documented [40,41], and we identified *O. viverrini *ORFs encoding membrane and secreted proteins, some of which were clearly more similar to vertebrate than to invertebrate homologues (Table 3). Transforming growth factor-beta (TGF-β) regulates cell growth and differentiation and is acquired on the cell surface by specific TGF-β receptors [42]. An ORF encoding a member of the TGF-β receptor type Ib family was identified in *O. viverrini*. The ORF included a 28 amino acid insertion absent from other type I TGF-β receptors, except for TR1 from the hydatid tapeworms of the genus *Echinococcus *(also members of the phylum Platyhelminthes) [43]. However, these two insertions did not share sequence identity (Figure 6A). Unlike many of the ESTs identified for which the closest homologues were from parasitic trematodes, the *O. viverrini *TGF-β receptor type I was divergent from SmRK-I of *S. mansoni *[44] and instead grouped more closely with proteins from *Echinococcus multilocularis *and from parasitic and free-living nematodes (Figure 6B). In pairwise sequence comparisons, however, the *O. viverrini *partial ORF was more similar to pig and macaque sequences (44% identity over 181 amino acids) than it was to *Echinococcus *TR1 (40% over 180 residues) or SmRK1 (40% over 182 residues). SmRK-I is known to bind to human TGF-β [40], suggesting that the *O. viverrini *receptor might also bind host growth factors for maturation and reproduction. Another OvAE encoding a protein which is potentially involved in the acquisition of host signals (and subsequent signaling) for growth and development was a fibroblast growth factor (FGF) receptor substrate 2. Parasitic flatworms induce fibrosis (and FGF) [45], and the parasites might acquire and utilize the host FGF that they induce for development and reproduction. Indeed, schistosomes are dependent upon FGF and transferrin for growth and maturation *in vitro *[46]. Of the sequences presented in Table 3, another OvAE which shared greatest identity with vertebrate homologues, encoded for calumenin, an EF-hand calcium binding protein localized to the secretory pathway. Calumenin is an inhibitor of the gamma-carboxylation system [47] and is expressed in thrombin-activated thrombocytes. It has a modulating effect on the organization of the actin cytoskeleton and may be involved in the pathophysiology of thrombosis or in wound healing [48]. The predicted calumenin of *O. viverrini *was most similar to rat and frog orthologues/paralogues, suggesting that it might interact with actin on the surface of host cells which are damaged during parasite feeding and migration.

### Molecules associated with cancer?

*O. viverrini *is the major cause of CCA in South-East Asia [1]. The molecular mechanisms underlying induction of *O. viverrini*-induced CCA are thought to be multi-factorial (reviewed in [49]), but recent evidence suggests that *O. viverrini *secretes mitogenic proteins into host tissues [1,8]. OvAEs encoding secreted proteins with prospective mitogenic activity were identified in the EST dataset. Of note, first, progranulin (*pgrn*) is a pluripotent secreted growth factor that mediates cell cycle progression, cell motility [50] and wound repair [51]. We identified an OvAE (OvAE1732) that shared sequence identity with *pgrn *(data not shown). Of particular importance is that *pgrn *has been implicated in regulating the proliferation of tumour cells, and its expression is up-regulated in more aggressive cancers (reviewed in [50]). The kallikrein-like serine proteases are another family of enzymes whose over-expression has been linked to cancer. The expression of some kallikreins in prostate cells leads to changes indicative of an epithelial to mesenchymal transition, an important process in cancer progression [52]. An OvAE (OvAE1918) with sequence identity to kallikrein-like secreted proteases is present in the new *O. viverrini *gene catalogue. Phospholipase A2 (PLA-2) regulates the provision of arachidonic acid to both cyclooxygenase- and lipoxygenase-derived eicosanoids (reviewed by [53]), and the upregulation of cyclooxygenase-2 is thought to be an important feature of cholangiocarcinogenesis in both humans and experimental rodent models [49,54,55]. We identified an OvAE (OvAE1644) that encodes a secreted PLA-2 which shared greatest sequence identity with PLA-2 from venom of *Heloderma *(Gila monster) and an EST from *C. sinensis *(Table 3). Parasites utilize secreted serine proteases [56] and PLA-2s [57] to invade host tissues, and homologues of these proteins (and granulin) are potentially secreted by *O. viverrini *into host tissues where they might promote cell proliferation, mutagenesis and ultimately carcinogensis. Ongoing studies in our laboratories are now focused on the physiological roles of these putative carcinogens in the host-parasite relationship and in cholangiocarcinogenesis induced by *O. viverrini *infection.

### Potential vaccines

Digenean flukes develop through a series of morphologically and developmentally discrete stages within their mammalian hosts, and each stage can be expected to display a characteristic transcritpome, confounding efforts to develop new control measures. Adult parasitic flukes are bound by an outer epithelial tegument, a structure that is widely regarded as the most vulnerable target for vaccines and drugs [32]. Homologues and orthologues of vaccine antigens identified in the tegument (and other structures from larval stages) of other flatworms were identified in the *O. viverrini *dataset (Table 4). Of particular note were the membrane spanning proteins, including an orthologue of the protective tetraspanin from *S. mansoni*, *Sm*-TSP-2 [32,33] (Figure 5) and the 22.6 kDa family of antigens from the schistosome tegument [58]. Homologues of gut proteases used by blood-feeding helminths to digest their blood-meal were identified from *O. viverrini*, including cathepsin D-like aspartic proteases [59,60], 11 distinct papain-like cysteine proteases [61-63] and the neutral protease, calpain, which associates with the inner tegument of schistosomes [64]. Other potential immunogens include lipid-binding proteins which are efficacious vaccines in the rabbit model against the western liver fluke, *Fasciola hepatica*, including saposin-like proteins [65] and fatty acid-binding proteins [66,67].

**Table 4:** *Opisthorchis viverrini *ESTs with sequence identity to mRNAs encoding proteins efficacious as vaccines against other flatworm and nematode parasites.

**Predicted protein family**	**Vaccine antigen and helminth targeted**	**References**
Aspartic protease	APR-1 for hookworm; cathepsin D for *Schistosoma japonicum*	[59, 60]
Glutathione-*S*-transferase	*Ac*-GST-1 for hookworm; bilvax for schistosomes	[79, 80]
Cysteine protease	TSBP for *Haemonchus contortus*; *Ac*CP1 for hookworm; Cathepsins L1 and L2 for *Fasciola hepatica*	[61–63]
Tetraspanin	TSP-1, TSP-2 and Sm23 for *Schistosoma mansoni*	[33, 81]
Pathogenesis related protein	ASP-2 for hookworm; ASP-1 for *Onchocerca volvulus*	[82, 83]
calpain	Smp80 for schistosomes	[64]
Fatty acid binding protein	Sm14 for *S. mansoni *and *F. hepatica*	[66, 67]
Saposin-like protein	FhSAP-2 for *F. hepatica*	[65]
14-3-3	Sm14-3-3 for schistosomes	[84]
22.6 (unknown function)	Sm22.6 for *S. mansoni*	[58]

## Conclusion

This report provides the first description of gene discovery for the liver fluke *O. viverrini*. Infection with *O. viverrini *is an important tropical health issue, but even more important and enigmatic is that chronic *O. viverrini *infection leads to the development of CCA. Indeed, there is no stronger link between a human parasite and cancer than that between *O. viverrini *and CCA [68]. The new gene catalogue for *O. viverrini *represents the largest EST dataset in the public domain for any species of liver fluke, and provides a platform for explorations into the molecular basis of host-helminth parasite interactions. We [1] and others [8] are interested in the molecules secreted into host tissues by *O. viverrini *that induce hyper-proliferation of biliary cells which can subsequently undergo malignant transformation. Given the number of *O. viverrini *ESTs sequenced herein, it is possible that mRNAs corresponding to these parasite mitogens are already present in the current dataset. Proteomic analysis of proteins secreted by adult *O. viverrini *maintained *in vitro *also is underway in our laboratories, and linking peptide sequences to corresponding mRNAs can be expected to be facilitated by this gene discovery program [12]. Finally, this gene discovery information for *O. viverrini *should expedite molecular studies of cholangiocarcinogenesis and accelerate research focused on developing new interventions, drugs and vaccines, to control *O. viverrini *and related flukes.

## Methods

### Parasite material

Adult *O. viverrini *were collected from experimentally infected hamsters (*Mesocricetus auratas*) maintained at the animal facility of the Khon Kaen University Faculty of Medicine. Protocols approved by the Khon Kaen University Animal Ethics Committee were used for all animal research conducted in this study. Briefly, metacercariae of *O. viverrini *were collected from naturally infected cyprinoid fish by pepsin digestion. Metacercariae (100 per hamster) were administered intragastrically to hamsters. Hamsters were euthanazed 6 weeks after inoculation, and adult worms were flushed with saline from the bile ducts [69]. Worms were washed extensively with sterile phosphate-buffered saline (pH 7.2), after which they were snap frozen and stored in liquid nitrogen or employed immediately as the source of fluke RNA.

### Construction and mass excision of cDNA library

Total RNA from adult *O. viverrini *was extracted using Trizol (Invitrogen), following the manufacturer's instructions. Ten μg of *O. viverrini *total RNA was used as a template for the synthesis of double-stranded cDNA using the SMART cDNA kit (BD Bioscience), after which the cDNA modified with adapters was cloned into the *Sfi *I site of the pTriplEx2 plasmid (BD Bioscience) and packaged into λ arms. The titer and percentage of recombinant phages in the library were determined using the protocols recommended by the manufacturer. *Escherichia coli *strain BM25.8 cells were transduced with recombinant phage, from which the excision of the pTriplEx phagemid library was accomplished.

### Clone selection, sequencing and annotation

Five thousand clones were randomly selected from the phagemid library and grown overnight in Luria Bertani (LB) broth supplemented with ampicillin to a final concentration of 25 μg/ml. Overnight cultures were shipped at 4°C in LB broth/ampicillin to the University of Melbourne (Department of Veterinary Science). The sequencing was performed by AgGenomics Inc., Australia, using a 3730xl DNA analyzer (Applied Biosystems). The TempliPhi™ DNA Sequencing Template Amplification system (GE Healthcare) was used to sequence each clone using the 5'λ TriplEx2 sequencing primer.

### Bioinformatic analyses

Edited sequences were condensed into contigs or singletons using TGICL [70] with the default parameters of 40 bp overlap, a minimum of 95% identity and a 30 bp maximum mismatched overhang. Sequences of less than 100 nt were discarded. Sequences were named using the same convention as that used for the human blood fluke, *Schistosoma mansoni *[13]; OvAE for *O*. *viverrini *Assembled EST. Sequences were compared with those available in the NCBI non-redundant protein and nucleotide databases using BLASTx and BLASTn. searches, respectively in October 2006. The dbEST database was queried using BLASTn and tBLASTx searches. BLAST alignments with an *E*-value of ≤ 1.0 × 10^-5 ^were reported. OvAEs were functionally categorized by querying a local copy of the Gene Ontology (GO) database [71] (downloaded November, 2006) with an *E*-value cutoff of 1.0 × 10^-5^. All ESTs from *C. sinensis *[9] and *Schistosoma japonicum *[11,12] were downloaded from NCBI [72], and the same methodology was used to derive ontology classifications for the *C. sinensis *ESTs. ORF predictions were performed using GENSCAN [73] using the HumanIso parameter set. Signal sequence prediction was accomplished using SignalP 3.0 [74], incorporating both hidden Markov models and neural networks. Positive signal sequence predictions from either method and positive signal anchor predictions using Markov models were reported. Predictions of transmembrane domains were conducted using TMPred [75]. All multiple sequence alignments were carried out using ClustalW. Clan and family assignments of proteolytic enzymes were analyzed via the MEROPS protease database [76]. Putative phosphorylation sites were predicted using the NetPhos 2.0 server [77].

### Phylogenetic trees

Multiple sequence alignments were assembled using ClustalW. Only regions which completely overlapped with partial ORFs of *O. viverrini *ESTs were used for tree construction. Alignments were imported into PAUP version 4.0 beta [78] to construct trees using the neighbour joining and maximum parsimony methods. Robustness was assessed by bootstrap analysis using 100 replicates. Clades with more than 50% support were denoted with bootstrap values on the branches.

### Cross-taxon similarity analysis

OvAEs were compared with all entries for other organisms in the NCBI dbEST database using tBLASTx. The highest BLAST scores (above a cut-off value of 50) were used to generate SimiTri plots [20] using software developed in-house (J. Mulvenna, unpublished).

## Authors' contributions

TL, PP and JM generated and analyzed the data and contributed to drafting of the ms. BS provided parasite material, helped conceive the study and edited the drafted ms. MS and MJS provided technical assistance and edited the drafted ms. RBG facilitated interactions with the sequencing unit and edited the drafted ms. PB helped conceive the study, supervised the research and helped draft the ms. AL helped conceive the study, supervised the research, and took the lead on drafting the ms. All authors read and approved the final ms.

## Supplementary Material

Additional file 1 *Opisthorchis viverrini *sequences that had homologues in the parasitic flukes, *Clonorchis sinensis *and *Schistosoma japonicum*, but not in the free-living platyhelminth, *Schmidtea mediterranea*. Click here for file
