# Supplementary material for: Gene discovery for the carcinogenic human liver fluke, Opisthorchis viverrini
Source: BMC Genomics. 2007 Jun 22;8:189. doi: 10.1186/1471-2164-8-189 (PMC1913519; doi:10.1186/1471-2164-8-189)
Supplement: Additional file 1 — Opisthorchis viverrini sequences that had homologues in the parasitic flukes, Clonorchis sinensis and Schistosoma japonicum, but not in the free-living platyhelminth, Schmidtea mediterranea. [file 1471-2164-8-189-S1.doc]

| **Clone** | **Closest homologue (nr)** |
| --- | --- |
| OvAE82 | SJCHGC01866 protein [Schistosoma japonicum] UPAR_LY6 domain |
| OvAE102 | myoglobin [Clonorchis sinensis]  Table S1.  *Opisthorchis viverrini* sequences that had homologues in the parasitic flukes, *Clonorchis sinensis* and *Schistosoma japonicum*, but not in the free-living platyhelminth, *Schmidtea mediterranea*. |
| OvAE112 | unknown |
| OvAE120 | unknown |
| OvAE122 | myoglobin [Clonorchis sinensis] |
| OvAE137 | myoglobin [Clonorchis sinensis] |
| OvAE147 | unknown |
| OvAE150 | myoglobin [Clonorchis sinensis] |
| OvAE153 | calcium binding protein [Clonorchis sinensis] |
| OvAE169 | SJCHGC08380 protein [Schistosoma japonicum] similar to Notch3 isoform 1 [Danio rerio] (evalue 0.53) |
| OvAE187 | Fatty acid-binding protein type 3 [Fasciola hepatica] |
| OvAE209 | myoglobin [Clonorchis sinensis] |
| OvAE218 | egg protein [Clonorchis sinensis] |
| OvAE221 | unknown |
| OvAE252 | SJCHGC04453 protein [Schistosoma japonicum] DM9 Repeats - putative Fasciola/Schistosoma cross-reactive protein [Fasciola hepatica] |
| OvAE278 | SJCHGC09766 protein [Schistosoma japonicum] |
| OvAE320 | legumain [Opisthorchis viverrini] |
| OvAE347 | similar to GenBank Accession Number U54584 histone H1 in Schistosoma |
| OvAE407 | unknown |
| OvAE438 | unknown |
| OvAE480 | novel transposon [Danio rerio] |
| OvAE494 | similar to GenBank Accession Number U54584 histone H1 in Schistosoma |
| OvAE496 | unknown |
| OvAE506 | unknown |
| OvAE515 | SJCHGC03431 protein [Schistosoma japonicum] COG1917 domain - Uncharacterized conserved protein, contains double-stranded beta-helix domain [Function unknown] |
| OvAE541 | glutathione S-transferase [Opisthorchis viverrini] |
| OvAE554 | vitelline B precursor protein [Opisthorchis viverrini] |
| OvAE565 | unknown |
| OvAE576 | SJCHGC06065 protein [Schistosoma japonicum] possible NADH:ubiquinone oxidoreductase |
| OvAE587 | vitelline B precursor protein [Opisthorchis viverrini] |
| OvAE598 | SJCHGC02380 protein [Schistosoma japonicum] possibly similar to ficolin 4 |
| OvAE614 | SJCHGC03431 protein [Schistosoma japonicum] COG1917 domain - Uncharacterized conserved protein, contains double-stranded beta-helix domain [Function unknown] |
| OvAE624 | polyprotein [Schistosoma japonicum] |
| OvAE654 | COS41.3 [Ciona intestinalis] |
| OvAE672 | vitelline B precursor protein [Opisthorchis viverrini] |
| OvAE673 | legumain [Opisthorchis viverrini] |
| OvAE685 | unknown |
| OvAE696 | unknown |
| OvAE703 | unknown |
| OvAE711 | egg protein [Clonorchis sinensis] |
| OvAE716 | SJCHGC09493 protein [Schistosoma japonicum] contains Baculoviral inhibition of apoptosis protein repeat domain; inhibitor of apoptosis protein [Schistosoma japonicum] |
| OvAE717 | egg protein [Clonorchis sinensis] |
| OvAE722 | cysteine protease [Opisthorchis viverrini] |
| OvAE731 | unknown |
| OvAE759 | unknown |
| OvAE774 | calcium binding protein [Clonorchis sinensis] |
| OvAE778 | unknown |
| OvAE815 | similar to GenBank Accession Number M94390 HEXBP DNA binding |
| OvAE816 | SJCHGC04841 protein [Schistosoma japonicum] |
| OvAE845 | calcium binding protein [Clonorchis sinensis] |
| OvAE869 | unknown |
| OvAE889 | SJCHGC09643 protein [Schistosoma japonicum] |
| OvAE934 | unknown [Clonorchis sinensis] possibly similar to anti-inflammatory protein 16 [Schistosoma mansoni] |
| OvAE936 | V-ATPase G subunit [Clonorchis sinensis] |
| OvAE937 | V-ATPase G subunit [Clonorchis sinensis] |
| OvAE944 | similar to GenBank Accession Number L24368 ribosomal protein |
| OvAE953 | CD63-like protein Sm-TSP-2 [Schistosoma mansoni] |
| OvAE968 | tegumental protein 22.3 kDa [Clonorchis sinensis] |
| OvAE982 | legumain [Opisthorchis viverrini] |
| OvAE990 | myoglobin [Clonorchis sinensis] |
| OvAE1020 | myoglobin [Clonorchis sinensis] |
| OvAE1026 | unknown |
| OvAE1034 | myoglobin [Clonorchis sinensis] |
| OvAE1046 | unknown |
| OvAE1082 | egg protein [Clonorchis sinensis] |
| OvAE1114 | unknown |
| OvAE1151 | myoglobin [Clonorchis sinensis] |
| OvAE1169 | RNA polymerase B transcription factor 3 [Schistosoma japonicum] |
| OvAE1211 | SJCHGC07206 protein [Schistosoma japonicum] Dynein_light, Dynein light chain type 1 – tegumental antigen Sm20 [Schistosoma japonicum] |
| OvAE1242 | lactate dehydrogenase [Clonorchis sinensis] |
| OvAE1349 | unknown |
| OvAE1391 | unknown |
| OvAE1402 | unknown |
| OvAE1403 | unknown |
| OvAE1408 | SJCHGC01393 protein [Schistosoma japonicum] weak homology to ATPase class I type 8B member 4 isoform 1 [Rattus norvegicus] |
| OvAE1433 | unknown |
| OvAE1464 | SJCHGC09800 protein [Schistosoma japonicum] |
| OvAE1474 | SJCHGC02820 protein [Schistosoma japonicum] similar to brain cell membrane protein 1 [Pan troglodytes] (evalue 0.99) |
| OvAE1491 | unknown |
| OvAE1496 | similar to GenBank Accession Number M94390 HEXBP DNA binding |
| OvAE1517 | vitelline B precursor protein [Opisthorchis viverrini] |
| OvAE1547 | unknown |
| OvAE1573 | myoglobin [Clonorchis sinensis] |
| OvAE1579 | unknown |
| OvAE1580 | SJCHGC01393 protein [Schistosoma japonicum] weak homology to ATPase class I type 8B member 4 isoform 1 [Rattus norvegicus] |
| OvAE1585 | unknown [Clonorchis sinensis] possibly similar to anti-inflammatory protein 16 [Schistosoma mansoni] |
| OvAE1587 | vitelline B precursor protein [Opisthorchis viverrini] |
| OvAE1588 | myoglobin [Clonorchis sinensis] |
| OvAE1602 | similar to GenBank Accession Number U54584 histone H1 in Schistosoma |
| OvAE1607 | egg protein [Clonorchis sinensis] |
| OvAE1619 | calcium binding protein [Clonorchis sinensis] |
| OvAE1634 | NADH dehydrogenase subunit 4 [Paragonimus westermani] |
| OvAE1649 | SJCHGC03047 protein [Schistosoma japonicum] |
| OvAE1658 | SJCHGC01866 protein [Schistosoma japonicum] UPAR_LY6 domain |
| OvAE1693 | SJCHGC03431 protein [Schistosoma japonicum] COG1917 domain - Uncharacterized conserved protein, contains double-stranded beta-helix domain [Function unknown] |
| OvAE1771 | SJCHGC02820 protein [Schistosoma japonicum] similar to brain cell membrane protein 1 [Pan troglodytes] (evalue 0.99) |
| OvAE1772 | lactate dehydrogenase [Clonorchis sinensis] |
| OvAE1775 | clonorporin 1 [Clonorchis sinensis] |
| OvAE1784 | fatty acid binding protein 15 [Schistosoma bovis] |
| OvAE1793 | unknown |
| OvAE1824 | legumain [Opisthorchis viverrini] |
| OvAE1877 | SJCHGC06570 protein [Schistosoma japonicum] contains Immunoglobulin domain - |
| OvAE1911 | lactate dehydrogenase [Clonorchis sinensis] |
